# A Feasibility Study of Fricke Dosimetry as an Absorbed Dose to Water Standard for ^192^Ir HDR Sources

**DOI:** 10.1371/journal.pone.0115155

**Published:** 2014-12-18

**Authors:** Carlos Eduardo deAlmeida, Ricardo Ochoa, Marilene Coelho de Lima, Mariano Gazineu David, Evandro Jesus Pires, José Guilherme Peixoto, Camila Salata, Mario Antônio Bernal

**Affiliations:** 1 Laboratório de Ciências Radiológicas, LCR-IBRAG-UERJ, Rio de Janeiro, RJ, Brazil; 2 Laboratório Nacional de Metrologia das Radiações Ionizantes, LNMRI-IRD, Rio de Janeiro, RJ, Brazil; 3 Instituto de Física Gleb Wataghin, Universidade Estadual de Campinas, Campinas, SP, Brazil; University of Nebraska Medical Center, United States of America

## Abstract

High dose rate brachytherapy (HDR) using ^192^Ir sources is well accepted as an important treatment option and thus requires an accurate dosimetry standard. However, a dosimetry standard for the direct measurement of the absolute dose to water for this particular source type is currently not available. An improved standard for the absorbed dose to water based on Fricke dosimetry of HDR ^192^Ir brachytherapy sources is presented in this study. The main goal of this paper is to demonstrate the potential usefulness of the Fricke dosimetry technique for the standardization of the quantity absorbed dose to water for ^192^Ir sources. A molded, double-walled, spherical vessel for water containing the Fricke solution was constructed based on the Fricke system. The authors measured the absorbed dose to water and compared it with the doses calculated using the AAPM TG-43 report. The overall combined uncertainty associated with the measurements using Fricke dosimetry was 1.4% for k = 1, which is better than the uncertainties reported in previous studies. These results are promising; hence, the use of Fricke dosimetry to measure the absorbed dose to water as a standard for HDR ^192^Ir may be possible in the future.

## Introduction

High dose rate brachytherapy (HDR) using ^192^Ir is well accepted as an important treatment option for cancer patients and thus requires an accurate dosimetry standard. A dosimetry standard for the direct measurement of the absolute absorbed dose (herein, referred to as ‘dose’) to water for this particular source type is currently not available. The AAPM TG-43 Report [1] and its update [2] outline the accepted protocol for determining the dose to water based on an air kerma strength (*S_k_*) measurement. The dose to water conversion is performed via the dose rate constant *Λ*, which converts the air-kerma strength to the dose to water, and several relative correction factors, which account for scatter, attenuation, and anisotropy of the dose distribution, among other effects [2]. The main concern regarding this method is that it is not a direct measure of the dose to water and thus may induce high uncertainties.

In clinical practice, it is necessary to measure the dose to water. Two potentially useful approaches have been reported. The first approach was developed by Sarfehnia et al. (2007) [3] using a water-based calorimeter with an uncertainty of 2.5% (*k* = 1) due to source self-heating, which affects the reading. Using a similar method, Sarfehnia and Seuntjens [4] and Sarfehnia et al. [5] recently reported an uncertainty reduction to 1.9% (*k* = 1). The second approach was developed by Austerlitz et al. [6] and uses Fricke dosimetry, with an overall uncertainty of 3.4% (*k* = 1). This high uncertainty is due to both the small dimensions of the irradiating device and the experimental procedures involved in this type of dosimetry. However, the results obtained with Fricke dosimetry must be improved to allow their use as a metrological reference.

Fricke dosimetry, also called ferrous sulfate dosimetry, is one of the most useful chemical dosimeters in existence. This dosimetry technique depends on the oxidation of ferrous ions (Fe^2+^) to ferric ions (Fe^3+^) by ionizing radiation. The increased concentration of ferric ions is measured spectrophotometrically at 304 nm. The Fricke dosimeter is 96% water by weight; therefore, its dosimetric properties are very similar to those of water. This dosimeter is used in a dose range of 5–400 Gy and for dose rates of up to 10^6^ Gy/s. The major disadvantages of Fricke dosimetry are its high sensitivity to impurities, which act as scavengers of the hydroxyl radicals generated by irradiation or as ferrous ion oxidants, resulting in a non-linear response and decreased system sensitivity when the oxygen present in the solution is depleted [6], [7], [8].

The main goal of this study was to develop a Fricke-based primary standard dosimetry for the dose to water measurements for HDR ^192^Ir sources. This paper presents important improvements compared with previous studies using this type of dosimetry [6], including a newly designed irradiation vessel, a new reading device, careful temperature control during irradiation and reading, and a more accurate calculation of the correction factors and uncertainties, resulting in a significant reduction of the overall uncertainty.

## Materials and Methods

### The Fricke system

The Fricke solution was prepared using chemicals of high purity, including ammonium iron (II) sulfate hexahydrate [(NH_4_)_2_Fe(SO_4_)_2_·6H_2_O] (99%), sodium chloride [NaCl] (99.5%), and sulfuric acid [H_2_SO_4_] (95.0–99.0%) (all purchased from MERCK-KGaA, Darmstadt, Germany), using a 1 L volumetric flask. First, 22 ml of sulfuric acid was diluted with 250 ml of Milli-Q water, and then 0.06 g of NaCl and 0.392 g of ferrous sulfate were added. The solution was added to the volumetric flask and diluted to the final volume of 1 L with Milli-Q water. The flask containing the Fricke solution was sealed and stored away from natural and artificial light sources for 24 h before use.

Ammonium sulfate and sodium chloride were weighed using a calibrated analytical Ainsworth model AA-200 balance with an accuracy of 0.0005 g. A density of 1.0230 g.cm^−3^ at 25°C was measured for the non-irradiated solution using a Densimeter Incoterm, calibrated at 22°C with a resolution of 0.0001 g.cm^−3^, which can be compared with the value of 1.0227 g.cm^−3^ at 25°C reported by Olszansky et al. [8]. Daily readings of optical density (*OD*), or absorbance, over a period of nine days using freshly made solutions showed no measurable differences compared with a month-old solution. Hence, a correction for fading was not considered.

The *OD*s of the Fricke dosimeter solutions were measured using a B-52 Micronal spectrophotometer with a digital LCD display at a wavelength of 304 nm, resolution of 1 nm, and photometric accuracy of 0.010 AU, which was tested with a set of traceable filters in the operational range of 190–1100 nm. The cuvette holder had four compartments for 1.0-cm-thick cuvettes. Because a thermal bath was unavailable, the temperature gradient was monitored until agreement was found between two thermal probes, which were calibrated against a mercury thermometer traceable to NIST, with 0.1°C resolution. The probes were mounted near the cuvette holder and outside the opening door. The cuvette compartment was manually moved in and out of the optical chamber, and the *OD* and temperature were continuously measured. The nominal dimensions of the three cuvettes were 1.0×1.0×4.5 cm^3^, and their optical path lengths were measured as 0.9995, 1.0005 and 0.9975 cm with an uncertainty of 0.0005 cm.

### The irradiation vessel design

**Figure 1:**
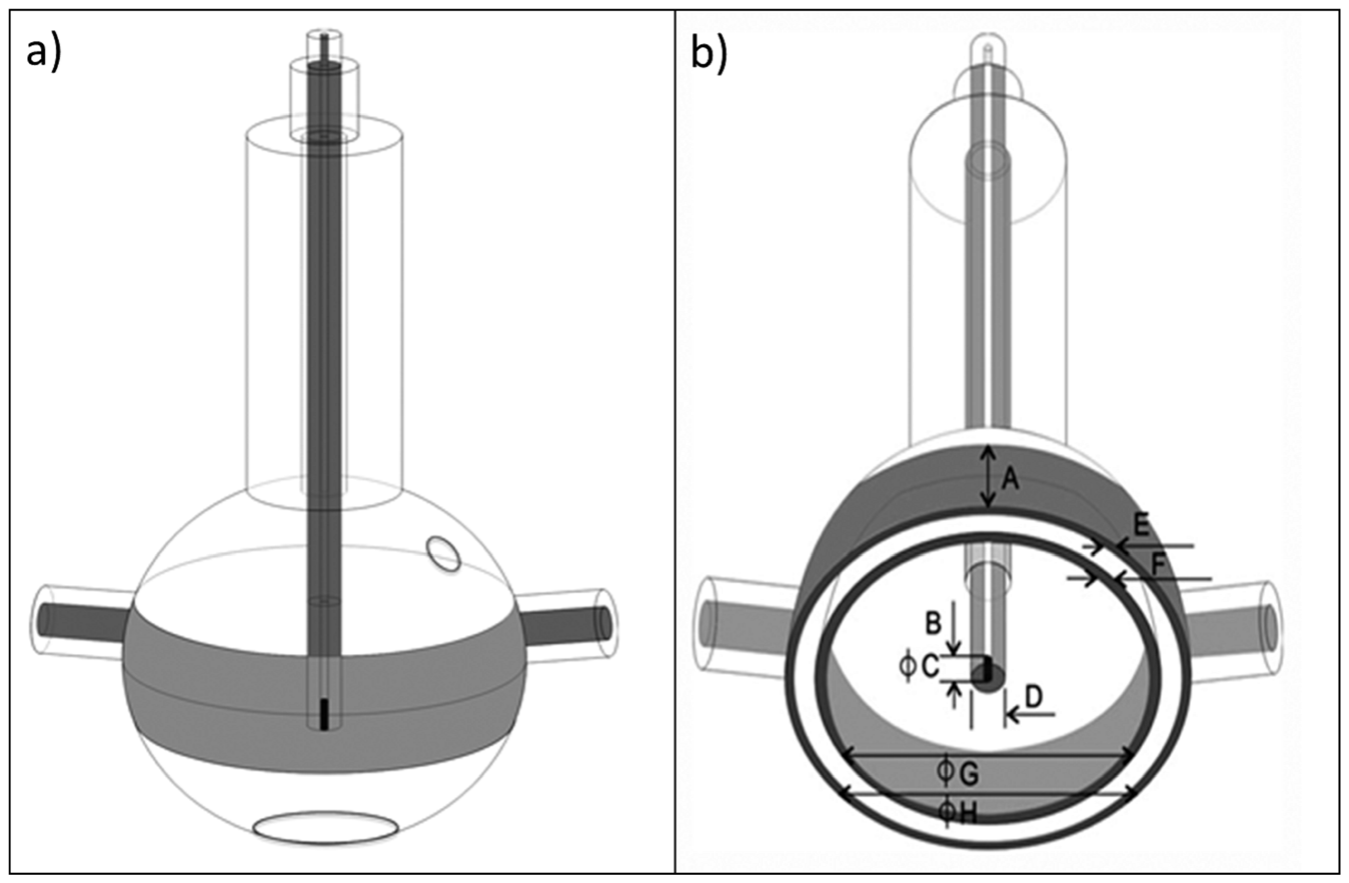
Irradiation vessel drawings. a) External view of the flask. The lateral openings are used to insert and remove the solution. b) Cross-sectional view of the flask, with external and internal dimensions. A: ring-shaped disc (18 mm); B: source length (3.50 mm); C: source diameter (0.60 mm); D: source-holder diameter (1.06 mm); E and F: PMMA wall thicknesses (1.27 mm and 1.62 mm); G and H: internal and external diameter of the vessel (45.09 mm and 54.19 mm).

Spherical vessels of polymethyl methacrylate (PMMA) were carefully handmade, and a PMMA tube was fixed at the top of the flask to allow the center of the source inside the catheter to coincide with the geometric center of the vessel, as shown in Fig. 1a. The irradiated solution volume was 8.0 cm^3^, which was sufficient to fill two cuvettes and obtain two readings for each irradiation. All of the dimensions are shown in Fig. 1b.

The effects due to possible chemical reactions between the FeSO_4_ solution and PMMA were tested over a long time period. A non-irradiated Fricke solution was observed to react with the PMMA during the first 24 hours, causing a significant increase in the optical density. However, no increase was noted after 48 hours. Five flasks were tested five times with non-irradiated solutions, and this short-term effect was only observed in new flasks. This reaction, as described by Morrison and Boyd [9] for organic esters, might be due to the acid hydrolysis of the ester groups of PMMA, which is a reaction that reaches equilibrium after a certain length of time. All the flasks used for the absorbed dose to water determinations were previously treated for more than 48 h with the Fricke solution. This procedure ensures thet all the flasks used will not react with the solution during the irradiation.

### Ionometric measurements for the determination of *D_w_*

The quantity *S_k_* for the ^192^Ir source was determined using a Farmer-type cylindrical chamber calibrated by the Brazilian Accredited Dosimetry Calibration Laboratory, as proposed by Marechal et al. [10] and Ferreira et al. [11] and recommended by the IAEA [12]. The microSelectron HDR ^192^Ir Alpha Omega source was used for the simulations and measurements. The source consists of an iridium metal (density of 22.42 g.cm^−3^) cylinder, measuring 0.60 mm in diameter and 3.50 mm in length. The iridium core is encapsulated in 316L stainless steel with a density of 7.99 g.cm^−3^. The outer diameter of the source is 1.10 mm, and the wall thickness is 0.19 mm. The cable is made of stainless steel with a diameter of 1.10 mm and an effective density of 4.81 g.cm^−3^. Because several irradiations and measurements were conducted on different occasions, the source activities are not specified.

Following the recommendations of Rivard et al. [2] and Melhus and Rivard [13], the quantity *S_k_* (with cGy.cm^2^_._h^−1^ = U) was then converted to the dose to water at 1 cm from the source center, *D_w_*, by Eq. (1):
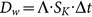
(1)where *Λ* is presently the most accurate dose rate constant value of 1.108 (±0.13%) cGy.h^−1^/U, which is specific for this source type, as reported by Daskalov et al. [14], and *Δt* is the time necessary to deliver the desired dose to the reference point. The dose to water calculated using this methodology was compared with the dose to water calculated using Fricke dosimetry.

### Irradiation and measurement procedures

The center of the spherical flask was filled with water, and the ring-shaped shell was filled with Fricke solution; the entire flask was placed in the center of the 30×30×30 cm^3^ water phantom. The irradiated solutions were inserted and extracted using a small Pyrex graduated pipette and were subsequently transferred to a quartz cuvette. The irradiation times used were calculated to deliver nominal doses ranging from 14 to 40 Gy. To minimize temperature gradients during irradiation, a thermoprobe monitored the temperature in the water phantom, and the irradiation was initiated only after the temperature stabilized.

Two cells were used to check the spectrophotometer response; the absorbances of pure water and of the non-irradiated Fricke solution were measured. Then, the absorbance of the irradiated and control solutions was measured. The temperatures measured during the spectrophotometer readings and during irradiation were used to correct the dose-induced change in *OD* using a reference temperature of 25°C. This relationship, initially described by Fregene [15] and modified by Olszanski et al. [8], is given in Eq. (2): 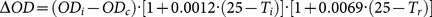
(2)where *OD_i_* and *OD_c_* are the optical densities of the irradiated and control solutions, respectively, *T_i_* is the temperature in °C of the Fricke solution during the irradiation, and *T_r_* is the temperature in °C of the Fricke solution during the spectrophotometer reading. The control samples were Fricke solutions that remained inside the vessel for the same amount of time as the irradiated solutions but were not irradiated.

### The determination of the absorbed dose to water using the Fricke dosimetry

As discussed by Klassen et al. (1999), the absorbed dose to the Fricke solution, *D_F_*, was obtained from the following equation: 
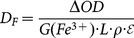
(3)where *ΔOD* is defined as the *OD* increase at 304 nm accounting for the temperature effect as determined by Eq. 2, *L* is the optical path length of the cuvette, *ρ* is the density of the Fricke solution (1.023 g cm^−3^) at 25°C, and *ε* is the molar linear absorption coefficient of the ferric ions (equal to 2174 M^−1^.cm^−1^ at 304 nm according to Klassen et al. [16]). *G(Fe^3+^)* is the radiation chemical yield of ferric ions (equal to 1.555±0.017×10^−6^ mol.*J^−1^*), which will be discussed further.

The quantity of the absorbed dose to water, *D_w_*, is derived from the absorbed dose to the Fricke solution, as proposed by Klassen et al. [16] and defined by Eq. (4):
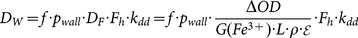
(4)where *D*_F_ is the absorbed dose in the Fricke solution, *f* is the dose conversion factor from the Fricke solution to water, *p_wall_* is the PMMA wall correction factor, *F_h_* is the homogeneity correction due to the volume-averaging effect as described by Ochoa et al. [17] and *k_dd_* is the correction factor due to the non-uniformity of the dose profile over the solution volume. These correction factors were calculated using the Monte Carlo method, as described below.

1) The correction for the volume-averaging effect, *F_h_*: The center of the solution volume was considered to be the reference point for dose calculations. This volume was divided into five equal, concentric spherical layers, and the absorbed dose was calculated for each layer and normalized to the dose of the central layer. The main components that influence the radial dose distribution are the self-attenuation of the Fricke solution and the non-uniformity of the photon fluence due to beam divergence, which causes a small dose gradient.

2) The non-water wall effect, *p_wall_*: This factor considers the influence of the PMMA wall from the vessel on the dose deposited in the Fricke solution compared with a vessel without walls. The *p_wall_* factor was calculated as the ratio of the absorbed dose to the Fricke solution in a volume detector without the PMMA walls to the absorbed dose obtained in the PMMA wall vessel.

3) The dose conversion factor from the Fricke solution to water, *f*: This factor is due to the difference in the dose deposited within the volume of Fricke solution compared with the dose that would be deposited in the same volume of water; this difference arises from the different radiation absorption characteristics and respective densities of the Fricke and water solutions. The *f* factor was calculated as a ratio, defined as the absorbed dose to water relative to the absorbed dose to the Fricke solution.

4) The correction factor for the non-uniformity of the dose profiles over the solution volume, *k_dd_*: This factor considers the magnitude of the anisotropy effect over 6 equally divided sectors around the source and along the axial direction. The central section was considered the reference point for the dose calculations.

### Monte Carlo simulations

The Monte Carlo particle-transport simulation code PENELOPE [18] was used to assess the data and necessary corrections. In all cases, several simulations were conducted with at least three different random number generator seeds, and the mean value was used. The simulations were performed on an Intel Pentium Dual core 3.4 GHz computer with 4.0 Gb RAM using the ^192^Ir bare spectra reported by Borg and Rogers [19].

To validate our calculation results, these values were compared with those of Borg and Rogers [19], Ma and Nahum [20] and Ma et al. [21], who used similar materials and geometry; in all three cases, very comparable results were obtained. The Fricke solution data obtained from the PENELOPE database (identification number 160, with a density of 1.024 g cm^−3^) were very close to the experimentally measured value of 1.023 g cm^−3^ for the solution in our measurements.

For the ^192^Ir simulations, 200 million primary photons were used, with cutoff transport energies of 1 keV for photons and 100 keV for electrons and a maximum step size of 0.01 cm for the Fricke solution. The time for each simulation was approximately 25 hours. The experimental vessel simulation was performed according to the measurements shown in Fig. 1b, using PMMA for all of the walls. The microSelectron source,described previously, was positioned in the center of the sphere, and the center of the vessel was placed at a depth of 10 cm in a 30×30×30 cm^3^ water phantom.

### Determination of the *G value*

Two different methodologies were used to determine the *G value* [*G(Fe^3+^)*]. The first consisted of the estimation of the energy-weighted *G value* from published values. A curve fitting was performed using the ionometric and calorimetric measurements reported by Fregene [15] and the calorimetric measurements reported by Klassen et al. [16]. It is important to highlight that the values obtained from Fregene [15] were reported in his paper without significant experimental detail. A *G value* was assigned for every 50 keV of energy in the energy interval from 1 to 900 keV. These values were weighted according to the photon fluence per MeV per 100 decays.

**Figure 2:**
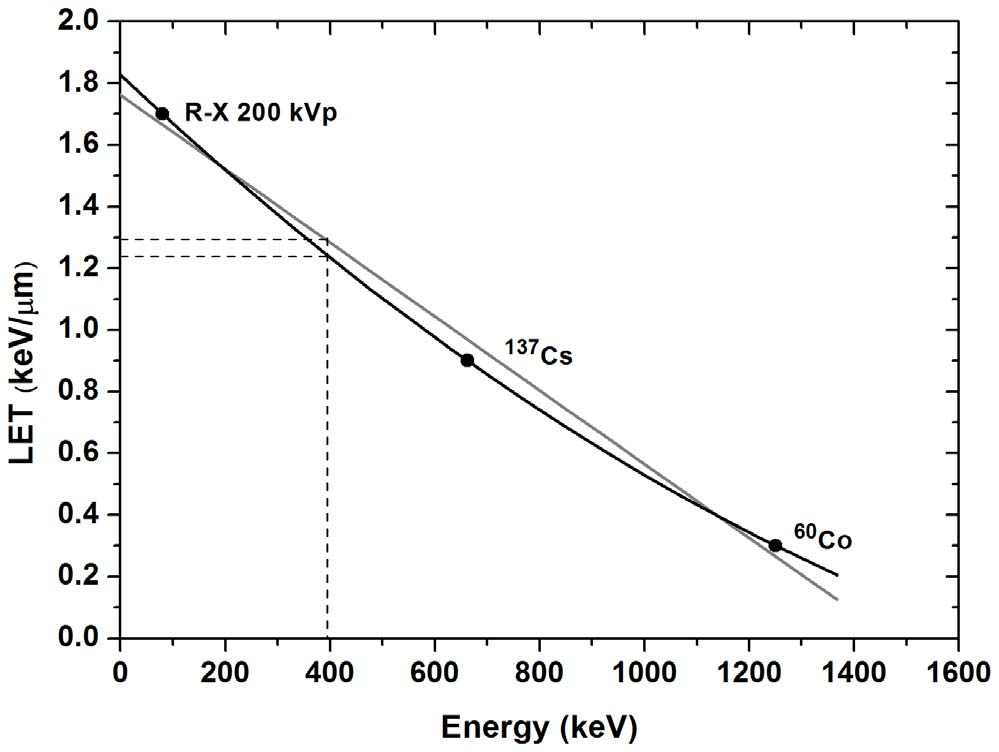
Energy versus LET. The interpolated LET value for the ^192^Ir average energy using published data [22], [23] and two different curve fittings.

In the second method, the *G value* was calculated based on the primary products. The radiation yield of ferric ions in a Fricke solution can be expressed in terms of the radiation yields of the primary products due to solution radiolysis. Thus, the *G values* were calculated using a fit of the *LET* values shown in Fig. 2 for 80 keV and ^6^°Co, both published by the ICRU [22], and for ^137^Cs, published by Meesungnoen et al. [23]. If the data are fitted using a first-order polynomial regression, the estimated *LET* value for ^192^Ir is 1.28 keV.µm^−1^; however, if the data are fitted with a second-order polynomial, the value is 1.237 keV.µm^−1^.

These values were used in the empirical formalism proposed by Meesungnoen et al. [23], given as equation 5, to calculate the *G value* in molecules per 100 eV for a given energy *x*:
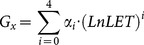
(5)where the coefficients 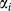 (*i* = 0–4) are used to express the *LET* variations for radicals and for the radiolysis of aqueous 0.4 M H_2_SO_4_ at room temperature. Table 1 presents the fitted coefficients for the aqueous 0.4 M H_2_SO_4_ at ambient temperature, as reported by Meesungnoen et al. [23].

**Table 1:** Numerical values of the coefficients for the aqueous 0.4 M H_2_SO_4_ used in the formalism proposed by Meesungnoen et al. [23].

Radicals	Coefficients				
	α_0_	α_1_ (x10^−2^)	α_2_ (x10^−2^)	α_3_ (x10^−2^)	α_4_ (x10^−3^)
G_H_	3.601	−13.53	−5.974	−1.929	−4.979
G_OH_	2.766	−18.80	−8.239	−2.127	−4.637
G_H2O2_	0.8438	5.682	2.169	0.6284	1.988

Because the Fricke solution was 96% water by weight, the primary products produced by the radiation were mostly those of water. This process, although considered approximate, was extensively discussed by Klassen et al. [16], where the *G values* for a Fricke solution were assumed to behave similarly to those for water.

## Results and Discussion

### Correction factors

The Monte Carlo (MC) calculations were used to determine the correction factors. For the experimental vessel geometry used in the present work, the MC factors in Eq. 4 are the following:

1) The correction for the volume-averaging effect, *F_h_*:

The absorbed dose calculated at the central layer was 0.4% lower than the average calculated dose of all layers. For this reason, a correction factor, *F_h_*, of 0.996±0.003 was considered.

2) The non-water wall effect, *p_wall_*:

The calculated factor was *p_wall_* = 0.999±0.004.

3) The dose conversion factor from the Fricke solution to water, *f*:

The obtained value was *f* = 1.004±0.003.

4) The correction factor for the non-uniformity of the dose profiles over the solution volume, *k_dd_*:

The obtained value was *k_dd_* = 1.000±0.002.

### G value

**Figure 3:**
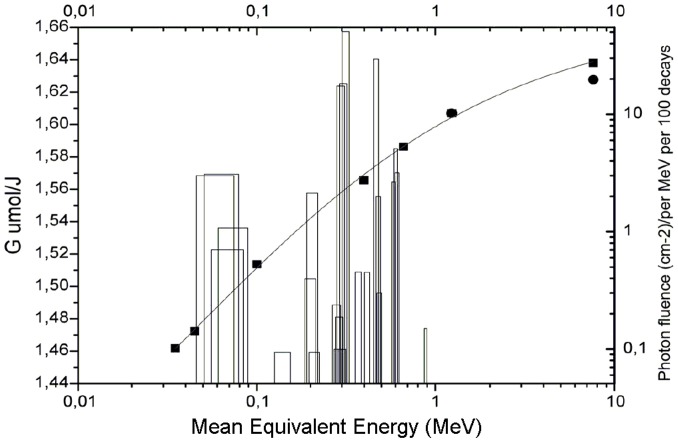
Estimation of the *G value*. The *G value* was estimated based on published values and the use of the energy weights for the ^192^Ir photon fluence calculated by MC simulations. Full circles are the values reported by Klassen et al. [16], full squares are those reported by Fregene [25], and the solid line is all of the data fitted in this work.

The *G value* obtained from the energy-weighted published values was 1.555±0.017×10^−6^ mol.J^−1^ and was used throughout this work. This value is comparable with the ionometric measurement data using dosimetry protocols reported by Franco et al. [24] (1.578±0.016×10^−6^ mol.J^−1^). The selection of the mean energy was not a critical issue for the semi-log plots used. The energy fluence for water was calculated by both Borg and Rogers [19] and in this work using Monte Carlo methods. Fig. 3 shows the plotted literature data used to calculate the *G value* [16], [25].

The *G(Fe^3+^)* obtained from the empirical formalism proposed by Meesungnoen et al. [23], based on the primary products and *LET* values, was found to be 15.123 mol/100 eV (1.567×10^−6^ mol.J^−1^) and 15.144 mol/100 eV (1.569×10^−6^ mol.J^−1^) for the first- and second-degree fits, respectively. This finding agrees with the *G value* determined above to within 1%.

### Absorbed dose to water measurements

**Figure 4:**
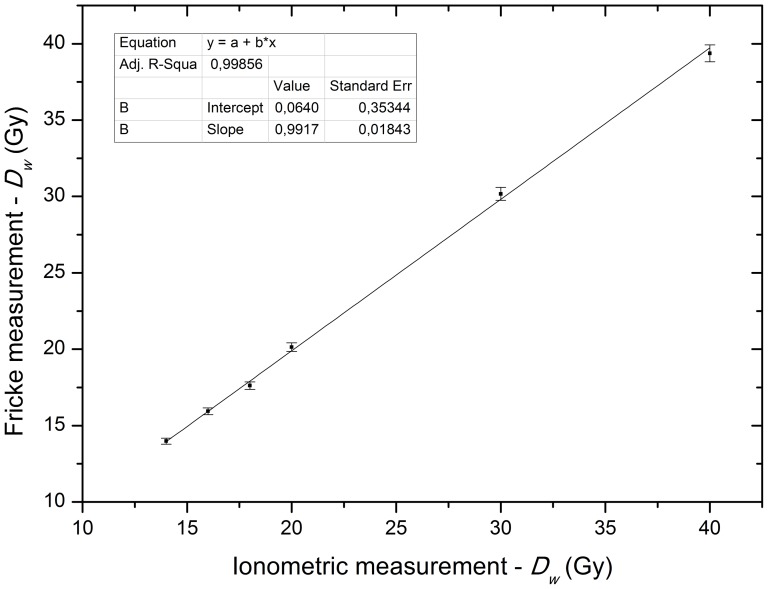
Fricke measurements. The absorbed dose to water values measured with Fricke dosimetry versus the nominal dose measured by a Farmer-type ionization chamber. The X-axis represents the measured absorbed dose values with the ionization chamber, and the Y-axis represents the measured absorbed dose values with the Fricke system with a total uncertainty of 1.4%, both for *k* = 1.

The results shown in Fig. 4 represent the average of three irradiations of the Fricke solution with two readings per irradiation per point and show the absorbed dose values ranging from 14.0 to 40.0 Gy.

The results presented here are consequences of a careful improvement of several aspects of our methodology compared with the previous work of Austerlitz et al. [6], such as the following:

- The overall dimension of the irradiating vessel (by increasing the radial distance between the source and the solution, the uncertainties due to mechanical tolerances and the dose gradient across the solution were reduced);
- The use of a calibrated thermistor in the spectrophotometer; and
- The use of PMMA, which made the construction of the vessel easier and, as discussed earlier, has no measurable effect on the solutions.

### Uncertainty budget

For this study, the nominal dose selected for the uncertainty calculations was 20 Gy. Table 2 lists all the sources of uncertainties involved in the experimental procedure to use Fricke dosimetry to measure the absorbed dose to water. The uncertainties are generally conservative and correspond to the upper limits. The uncertainties in all quantities and correction factors in Eq. 4 are indicated. As a result, the overall combined uncertainty, as described in detail in Table 2, was significantly reduced to 1.4% for k = 1 compared with those reported earlier by Austerlitz et al. [6].

**Table 2:** Uncertainty budget in the determination of *D_w_* using the Fricke solution.

Source of Uncertainty	Type A (%)	Type B (%)	Reference
**Irradiation Procedure**			
Dummy/real source position		0.1	
Transit time		0.016	
**Solution Specification**			
Molar extinction coefficient		0.35	[16]
Density	0.100	0.100	Manufacture
Source-solution distance	0.01	0.02	Manufacture
**Reading Process**			
Dose determination	0.48		Manufacture
Cuvette-light path	0.05	0.06	Manufacture
Instrument stability		0.10	
Instrument repeatability		0.10	
Wavelength bandwidth		0.01	[27]
Solution temperature	0.010	0.15	Manufacture
**Correction Factors**			
*G(Fe^3+^)* value		1.12	[15], [24], [28]
*p_wall_*	0.3	0.2	[26]
Volume averaging	0.2	0.2	[26]
*k_dd_*	0.1	0.2	[26]
Dose conversion factor for Fricke to water *f*	0.2	0.2	[26]
***Combined Standard Uncertainty (%)***	1.42		
***Expanded Uncertainty for k = 2.0 (%)***	2.84		

The type B uncertainties for the MC calculations were the most difficult to estimate, and they remain unclear in several papers. In a recent work, Wulff et al. [26] specifically addressed this issue, taking into account the various contributions related to the systematic uncertainties that are also in the present work, such as stopping power, spectrum, photon cross sections and transport parameters. Although we did not determine a specific analysis for our geometry, the final value of 0.2% reported by Wulff et al. [26] was adopted here.

## Conclusions

Chemical dosimetry using a standard FeSO_4_ solution in a containment vessel with a uniform geometry relative to the source has been shown to be a feasible option for the absorbed dose standard for HDR ^192^Ir sources. The overall uncertainty involving the vessel dimensions, wall thicknesses, dose calculation, wall attenuation, UV light band, source anisotropy, *G value* and source transit time was estimated to be less than 1.4% for *k* = 1.

A comparison of this study with different studies performed using absorbed doses derived from either a water-based calorimeter or air-kerma ionization chamber measurements would be very useful. The initial results of this work should be of primary interest to calibration laboratories as a means to establish a reference for the quantity absorbed dose to water and to enhance the traceability of methods that are presently not yet suitable for a clinical environment.

However, several improvements are necessary to obtain more reliable results, including better control of the temperature during the read-out process, small modifications of the vessel to make it easier to fill with Fricke solution, the use of a more precise spectrophotometer, and improvement of the *G value* calculation method to obtain superior uncertainty.
